# Restaurant managers’ readiness to maintain people’s healthy weight and minimise food waste in Japan

**DOI:** 10.1186/s12889-022-13274-x

**Published:** 2022-04-26

**Authors:** Rie Akamatsu, Nozomi Tonsho, Mika Saiki, Mihono Komatsu

**Affiliations:** 1 Faculty of Core Research, Natural Science Division, Ochanomizu University, Tokyo, Japan; 2 Former Graduate School of Humanities and Sciences, Ochanomizu University, Tokyo, Japan

**Keywords:** Restaurants, Public policy, Food waste, Obesity, Sustainable development

## Abstract

**Background:**

People who consume high amounts of ready-to-eat meals have a higher body mass index than those who do not. However, if customers adhere to eating proper amounts without restaurants reducing the portions, plate waste may occur. It is therefore incumbent upon restaurants to serve suitable meal portions to customers in the interests of their health and the environment. This study examined whether restaurants support Japan’s national goals of minimising food loss and waste and maintaining healthy body weight. Additionally, the characteristics of restaurant managers who display a willingness to meet these goals, were identified.

**Methods:**

An internet-based nationwide cross-sectional survey was conducted among restaurant managers in Japan in May 2019. The main outcome measured was readiness to take action toward maintaining healthy weight and minimising food loss and waste. Chi-square tests were performed to examine the characteristics of three groups, divided on the basis of their readiness to take action toward achieving these two goals. The group that displayed a higher readiness toward attaining both goals was examined using logistic regression analyses.

**Results:**

Of the 412 restaurant managers who responded, 387 (93.9%) were analysed. Few managers reported taking action toward maintaining healthy weight (*n* = 13, 3.4%) and minimising food loss and waste (*n* = 45, 11.6%). Two variables, ‘medium- or big-sized company’ and ‘referring to the United Nations’ Sustainable Development Goals for running their business’, were related to both higher readiness for maintaining healthy weight and minimising food loss and waste (odds ratio [OR] = 2.27, confidence interval [CI]: 1.11–4.62; OR = 4.06, CI: 2.39–6.88, respectively).

**Conclusions:**

Increasing recognition of the Sustainable Development Goals among restaurant managers is needed to increase their cooperation toward meeting Japan’s national goals.

## Background

Restaurants, including those offering takeout or delivery of ready-to-eat meals, are popular worldwide [1–5]. Japan is no exception—approximately 40% of men and 30% of women across all ages consume ready-to-eat meals more than once a week; moreover, the percentage was noted to be about 50% among men aged 20–50 [6]. Increased consumption of ready-to-eat meals leads to increased risk of overeating and unbalanced nutrition [2, 7]. According to a study that surveyed the energy content of restaurant meals in five countries—Brazil, China, Finland, Ghana, and India—the unadjusted mean energy content for all countries was 1317 kcal per meal in full-service restaurants and 809 kcal for fast food, and their energy densities were 1.98 kcal/g and 1.92 kcal/g, respectively, indicating high-fat meals [8]. Although the energy content of Japanese meals is lower, a study reported the mean content for the lunch menu at 935 kcal, which is higher than the recommended 650–850 kcal [9]. Adult Japanese men, who have a high propensity for enjoying ready-to-eat meals, have the highest obesity rate, of over 30%, compared to other groups in Japan. This is consistent with studies in other countries, which indicated that people who consume more ready-to-eat meals have higher body mass indexes than those who do not indulge in such meals [3–5, 10].

However, if customers know and adhere to appropriate meal quantities, the problem of plate waste may occur, which can lead to food waste. Food waste refers to a decrease in the quantity or quality of food resulting from decisions and actions by retailers, food service providers, and consumers [11]. The highest amount of food waste among business activities is generated by the restaurant industry in Japan; in 2017, the recycling rate of food waste was only 32% in the restaurant industry, as compared to 95% in food manufacturing companies [12]. Large portions are one of the reasons for plate waste [13] as well as the cause of overeating [14].

To solve these problems—reducing obesity and plate waste—the Japanese government has set goals for both maintaining healthy weight and minimising food waste, including plate waste. The former is one of the targets of the second Health Japan 21 (2013–2023) [15], while the latter forms part of the fourth Basic Programme for *Shokuiku* (food and nutrition education) Promotion (2021–2025) [16]. These challenges are not limited to Japan, but are prevalent worldwide. The United Nations released the Sustainable Development Goals (SDGs) in 2015, which included points related to these two issues: Goal 3 is to ensure healthy living and to promote well-being for all at all ages (Goal 3.4: ‘... to reduce by one third premature mortality from non-communicable diseases ...’), and Goal 12 is aimed at ensuring sustainable consumption and production patterns (Goal 12.3: ‘... to halve per capita global food waste ...’) [17]. To achieve these goals, food companies’ cooperation is needed. Although the importance of the accountability of food companies for creating healthy food environments has been advocated before [18], there has been increased emphasis on taking action toward creating a sustainable environment since the SDGs were released.

The restaurant industry can potentially contribute to solving these problems by not serving large portions to customers. Research has shown that reducing portion sizes can lead to reduced caloric intake and plate waste [19, 20]. If restaurants serve smaller portions, they can reduce plate waste and economize [21]. Thus, restaurants serving appropriate food portions would benefit no less than three sectors: public health (e.g., reducing obesity), environment (e.g., reducing food waste), and business (e.g., saving money). However, inadequate data exist regarding restaurant managers’ awareness of these goals and the actions that must be taken to achieve them. This study examined whether restaurants in Japan support the national goals of maintaining healthy weight and minimising food waste, and investigated the characteristics of restaurant managers with higher readiness to take action to contribute to the achievement of these goals.

## Methods

### Participants and procedures

This study was part of the Study for Building a Partnership with Food Companies for a Healthy Food Environment, a cross-sectional study conducted via an internet survey through a Japanese research company (Macromill Inc.) which has nationwide monitors; the study was conducted from 28 to 30 May 2019. Participants were individuals who managed restaurants and served lunches; restaurants that only served dinner were excluded. The focus was on lunch because dinner at restaurants in Japan is more likely associated with events; therefore, restaurant managers may find it difficult to determine individuals’ meal sizes at dinner. A total of 950 people met these criteria among the monitors. This study was approved by Ochanomizu University’s Research Ethics Board (No. 2019–3).

### Measures

In addition to age and sex, job-related details were also obtained, such as work experience (years) in the restaurant industry, the size of the company they managed [22], whether SDGs were taken into consideration in running the business, experience of studying nutrition, and the involvement of dietitians to support the operations of the restaurant or company. We prepared five options to inquire about their willingness to consider the SDGs in their operations.

Six items were used to determine the characteristics of the restaurants managed by the participants: whether the restaurant was for everyday use or only for events, the region in which the restaurant was located, the type of restaurant, the number of seats for patrons, average spending per customer, and the frequency of customer visits. If the participants managed multiple restaurants, they were requested to answer on behalf of the one with the highest lunch-time sales.

Participants were asked if their company or restaurant had taken action to achieve the two national goals—maintaining healthy weight and minimising food waste—using the following question: ‘Do you think your restaurant can contribute to achieving these goals? Please choose one of the five choices for each goal’. These five choices were ‘we have already started taking action’, ‘we plan to take action’, ‘we have not planned it yet but want to take action’, ‘this goal is difficult for us to take action on’, and ‘this goal is not related to us’.

### Statistical analysis

First, descriptive statistics were used to assess the characteristics of restaurant managers and their restaurants. After comparing the answers regarding readiness for the two national goals by using chi-square tests, the five choices for readiness were divided into two categories: higher readiness (we have already started taking action, we have planned to take action, and we have not planned yet but want to take action) and lower readiness (this goal is difficult for us to take action on and this goal is not related to us). Next, we identified four groups of readiness based on combinations of the two categories, after which the groups with one higher and one lower level of readiness were combined to form one ‘high and low’ group. Thus, three readiness groups were finally obtained: ‘both higher’, ‘high and low’, and ‘both lower’. We compared the characteristics of restaurant managers according to these three groups using chi-square tests. Finally, logistic regression analyses were conducted to examine the characteristics of the ‘both higher’ readiness group. The dependent variable was readiness to take action (1 = ‘both higher’; 0 = ‘other (high and low, both lower)’, and the independent variables were the characteristics of the restaurant managers. Univariate regressions were conducted in Model 1, and multiple regression was conducted in Model 2, adjusting for age, sex, and the restaurant’s location. Logistic regression analyses were performed after confirming that the sample included at least 10 events per variable [23]. All analyses were performed using IBM’s SPSS for Windows (version. 26.0, SPSS Inc.). The results were considered statistically significant if *P*-values were less than 0.05.

## Results

### Description of participants and their restaurants

A total of 412 restaurant managers participated in the survey (response rate 43.4%). As this study targeted everyday life, we excluded restaurants used for events (25 [6.1%] were excluded). Finally, data of 387 participants (93.9%) were analysed. Approximately 70% of the respondents were in their 40s or 50s, and more than 70% were men. Half of them had been working in the restaurant industry for more than 20 years, and more than 80% worked at small businesses with less than five employees. A total of 35.4% participants had experience of studying nutrition and only 6.6% employed dietitians. Only 2.8% took cognisance of the SDGs in the running of their enterprises and more than 70% answered ‘never heard about it’ (Table 1).

**Table 1:** Characteristics of the restaurant managers

	Total
Age range (years)	
20–29	3 (0.8)
30–39	41 (10.6)
40–49	126 (32.6)
50–59	141 (36.4)
≥ 60	76 (19.6)
Sex	
Female	101 (26.1)
Male	286 (73.9)
Experience of working in the restaurant industry (years)	
< 5	32 (8.3)
5–9	59 (15.2)
10–14	43 (11.1)
15–19	34 (8.8)
≥ 20	219 (56.6)
Size of the company managed ^a^	
Small	337 (87.1)
Medium/Large	50 (12.9)
Refer to SDGs for running business	
Refer	11 (2.8)
Want to refer but not doing now	21 (5.4)
Know about SDGs but do not plan to use them	24 (6.2)
Heard the name but do not know the content	42 (10.9)
Never heard about it	289 (74.7)
Experience of studying nutrition	
Yes	137 (35.4)
No	250 (64.6)
Involvement of dietitians in the store or company ^b^	
Yes	25 (6.6)
No	356 (93.4)

*N* = 387; *SDGs* Sustainable Development Goals

^a^Small, fewer than five employees; medium, less than 50-million-yen investment or capital or fewer than 100 employees; large, more than 50-million-yen investment or capital or more than 100 employees [22]

^b^*n* = 381

In terms of the characteristics of the restaurants managed by the participants: the restaurants were spread across the country but approximately 30% were in the Kanto region, which includes Tokyo. The type of restaurant varied: A total of 28.5% had 10–19 seats, and more than half reported their customers’ expenditure as lower than 1000 yen (USD 9.07). More than half of the respondents reported the frequency of customer visits to be more than a day per week (Table 2).

**Table 2:** Characteristics of the restaurants

	Total
Region of restaurant location in Japan^a^	
Hokkaido-Tohoku	51 (13.2)
Kanto	109 (28.2)
Chubu	71 (18.3)
Kinki	95 (24.5)
Chugoku-Shikoku	27 (7.0)
Kyushu	34 (8.8)
Type of restaurant^b^	
Casual dining	83 (21.4)
Specialised restaurant	113 (29.2)
Fast food	83 (21.4)
Cafe	77 (19.9)
Pub/bar	31 (8.0)
Number of seats^c^	
< 9	35 (9.1)
10–19	110 (28.5)
20–29	87 (22.5)
30–39	65 (16.8)
40–49	26 (6.7)
≥ 50	63 (16.3)
Expenditure per customer (yen)^d^	
≤ 999	214 (55.4)
1000–1499	115 (29.8)
≥ 1500	57 (14.8)
Frequency of customer visits	
Almost everyday	38 (9.8)
2–3 days per week	106 (27.4)
1 day per week	89 (23.0)
2–3 days per month	61 (15.8)
1 day per month	51 (13.2)
Fewer than 1 day per month	42 (10.9)

*N* = 387

^a^The six regions include the following prefectures: Hokkaido-Tohoku (Hokkaido, Aomori, Iwate, Miyagi, Akita, Yamagata, Fukushima), Kanto (Ibaragi, Tochigi, Gunma, Saitama, Chiba, Tokyo, Kanagawa), Chubu (Niigata, Toyama, Ishikawa, Fukui, Yamanashi, Nagano, Gifu, Shizuoka, Aichi), Kinki (Mie, Shiga, Kyoto, Osaka, Hyogo, Nara, Wakayama), Chugoku-Shikoku (Tottori, Shimane, Okayama, Hiroshima, Yamaguchi, Tokushima, Kagawa, Ehime, Kochi), Kyushu (Fukuoka, Saga, Nagasaki, Oita, Kumamoto, Miyazaki, Kagoshima, Okinawa)

^b^Casual dining includes ordinary restaurants, except speciality restaurants. Specialised restaurants are restaurants with one specialised menu, such as sushi or tempura, or other international cuisines such as Italian. Fast food includes Japanese fast food such as ramen, udon, and noodles. Cafe denotes shops that serve not only coffee but also light meals

^c^*n* = 386

^d^*n* = 386; (1000 yen = USD 9.07 as of 20 June 2021)

### Readiness to take action to achieve the national goals

**Fig. 1:**
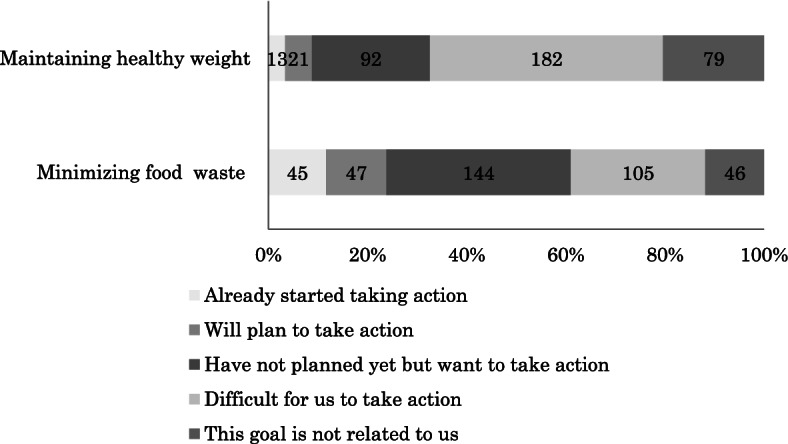
Readiness to take action to achieve the national goals. *N* = 387

Only a few managers reported having taken action to achieve the national goals of maintaining healthy weight (*n* = 13, 3.4%) and minimising waste (*n* = 45, 11.6%). As regards maintaining healthy weight, most managers chose the options ‘difficult for us to take action’ (*n* = 182, 47.0%) and ‘have not planned yet but want to take action’ (*n* = 144, 37.2%, *p* < 0.001; Fig. 1).

Based on the results, we generated two categories: *Higher Readiness* and *Lower Readiness*. A total of 32.6% (*n* = 126) formed the Higher Readiness group for maintaining healthy weight and 61.0% (*n* = 236) for minimising food waste. The percentages of the four groups resulting from combining these categories were 29.2% (*n* = 113) for both higher readiness groups and 35.7% (*n* = 138) for both lower readiness groups. Although 31.8% (*n* = 123) reported lower readiness for maintaining healthy weight and higher readiness for minimising food waste, the figure was only 3.4% (*n* = 13) for higher readiness for maintaining healthy weight and lower readiness for minimising food waste. These two groups were combined to ultimately obtain three readiness groups: *Both Higher Readiness* (*n* = 113, 29.2%), *High and Low Readiness* (*n* = 136, 35.1%), and *Both Lower Readiness* (*n* = 138, 35.7%; Table 3).

**Table 3:** Combinations of readiness to take action to achieve national goals

		Minimise food waste	
		Higher readiness (*n* = 236, 61.0%)	Lower readiness (*n* = 151, 39.0%)
Maintain healthy weight	Higher readiness (*n* = 126, 32.6%)	113 (29.2)	13 (3.4)
	Lower readiness (*n* = 261, 67.4%)	123 (31.8)	138 (35.7)

*N* = 387; n (%), percentage of the total number

‘Higher readiness’ includes ‘already started taking action’, ‘will plan to take action’, and ‘have not planned yet but want to take action’. ‘Lower readiness’ includes ‘difficult for us to take action’ and ‘this goal is not related to us’

### Characteristics of groups based on their readiness to take action

A comparison of the characteristics of the three readiness groups (Table 4) revealed only four items with significant differences: size of company, taking SDGs into account, experience of studying nutrition, and involvement of dietitians. There were more medium and big companies in the Both Higher Readiness group (*p* = 0.001). With regard to the group that referred to SDGs for running their businesses; fewer participants answered ‘never heard about it’ in the Both Higher Readiness group (52.2%) than other groups (high and low readiness = 80.9%, Both Lower Readiness = 87.0%, *p* < 0.001). There were 46.9% participants with experience of studying nutrition in the Both Higher Readiness group and approximately  30% in High and Low Readiness and Both Lower Readiness groups (*p* < 0.001). The percentages were low for hiring dietitians across all groups, but more managers in the Both Higher Readiness group employed dietitians (*p* = 0.002).

**Table 4:** Comparison of the characteristics of restaurant managers by readiness to take action ^a^

	Both higher	High & low	Both lower	*p*
	*n* = 113	*n* = 136	*n* = 138	
Age range (years)				
20–29	0 (0.0)	1 (0.7)	2 (1.4)	0.278
30–39	18 (15.9)	15 (11.0)	8 (5.8)	
40–49	36 (31.9)	45 (33.1)	45 (32.6)	
50–59	42 (37.2)	47 (34.6)	52 (37.7)	
≥ 60	17 (15.0)	28 (20.6)	31 (22.5)	
Sex				
Female	26 (23.0)	40 (29.4)	35 (25.4)	0.504
Male	87 (77.0)	96 (70.6)	103 (74.6)	
Experience of working in the restaurant industry (years)				
< 5	12 (10.6)	10 (7.4)	10 (7.2)	0.329
5–9	15 (13.3)	22 (16.2)	22 (15.9)	
10–14	14 (12.4)	19 (14.0)	10 (7.2)	
15–19	14 (12.4)	7 (5.1)	13 (9.4)	
≥ 20	58 (51.3)	78 (57.4)	83 (60.1)	
Size of company managed^b^				
Small	87 (77.0)	126 (92.6)	124 (89.9)	0.001
Medium/large	26 (23.0)	10 (7.4)	14 (10.1)	
Refer to SDGs for running business				
Refer	8 (7.1)	3 (2.2)	0 (0.0)	<.001
Want to refer but not doing now	17 (15.0)	4 (2.9)	0 (0.0)	
Know about SDGs but do not plan to use them	15 (13.3)	3 (2.2)	6 (4.3)	
Heard the name but do not know the content	14 (12.4)	16 (11.8)	12 (8.7)	
Never heard about it	59 (52.2)	110 (80.9)	120 (87.0)	
Experience of studying nutrition				
Yes	53 (46.9)	43 (31.6)	41 (29.7)	< 0.001
No	60 (53.1)	93 (68.4)	97 (70.3)	
Involvement of dietitians in the restaurant or company^ c^				
Yes	15 (13.3)	4 (3.0)	6 (4.5)	0.002
No	98 (86.7)	130 (97.0)	128 (95.5)	

*N* = 387; *SDGs* Sustainable Development Goals

^a^‘Both higher’ indicates higher readiness to take action toward both minimising food waste and maintaining healthy weight. ‘High & low’ indicates higher readiness to take action toward either minimising food waste or maintaining healthy weight and lower readiness on the other variable. ‘Both lower’ indicates lower readiness to take action toward both minimising food waste and maintaining healthy weight

^b^Small, fewer than five employees; medium, less than 50-million-yen investment or capital or less than 100 employees; large, more than 50-million-yen investment or capital or more than 100 employees [22]

^c^*n* = 381

Table 5 shows the odds ratios (ORs) of the characteristics of the Both Higher Readiness group. Only two items—size of company and referring to SDGs—remained significant characteristics of this group in the multiple regression. Restaurant managers managing medium or large companies who acknowledged the SDGs in their business operations were more likely to have higher readiness to both maintaining healthy weight and minimising food waste (OR = 2.27, confidence interval [CI]: 1.11–4.62; OR = 4.06, CI: 2.39–6.88, respectively).

**Table 5:** Odds ratios for characteristics of restaurant managers with higher readiness to take action toward both national goals^a^

	Readiness to take action		Univariate regression OR (95% CI)	Multiple regression^c^ OR (95% CI)
	Other^b^ *n* = 274	Both higher^b^ *n* = 113		
Experience of working in the restaurant industry (years)				
< 20	113 (41.2)	55 (48.7)	1	1
≥ 20	161 (58.8)	58 (51.3)	0.71 (0.43–1.16)	0.62 (0.36–1.06)
Size of company managed				
Small	250 (91.2)	87 (77.0)	1	1
Medium/Large	24 (8.8)	26 (23.0)	2.96 (1.60–5.51) **	2.27 (1.11–4.62) *
Refer to SDGs for running business^d^				
Never heard about it	230 (83.9)	59 (52.2)	1	1
Other	44 (16.1)	54 (47.8)	4.76 (2.90–7.83) ***	4.06 (2.39–6.88) ***
Experience of studying nutrition				
No	190 (69.3)	60 (53.1)	1	1
Yes	84 (30.7)	53 (46.9)	1.98 (1.25–3.13) **	1.38 (0.82–2.33)
Involvement of dietitians in the store or company^e^				
No	258 (96.3)	98 (86.7)	1	1
Yes	10 (3.7)	15 (13.3)	3.79 (1.60–8.95) **	1.63 (0.60–4.42)

*OR*, odds ratio, *CI* confidence interval

*N* = 387; n (%); *p < 0.05, ** *p* < 0.01, ***p < 0.001

^a^Dependent variable, readiness to take action (0 = other); independent variable, the characteristics of the restaurants’ managers. All models adjusted for age, sex, and the restaurants’ location

^b^‘Both higher’ indicates higher readiness to take action toward both minimising food waste and maintaining healthy weight. ‘Other’ indicates readiness to take action toward either minimising food waste or maintaining healthy weight and lower readiness toward the other variable, or lower readiness toward both

^c^Multiple regression: all variables were entered together as independent variables using forced entry

^d^SDGs, Sustainable Development Goals; others, heard the name but do not know the content, know about it but no plan to use it, want to refer to it but not now

^e^*n* = 381

## Discussion

This study examined whether restaurants in Japan support the national goals of maintaining healthy weight and minimising food waste, and investigated the characteristics of restaurant managers with a higher readiness to take action toward these goals. According to the results, very few restaurants have begun to take action to achieve these goals, and company size and reference to the SDGs for running their businesses were related to higher readiness to take action.

As habitual eating behaviour may impact health, we targeted restaurants for daily use, as evidenced by the results that the restaurants were more likely to have repeat customers more than 1 day per week and customers paid less than 1000 yen (USD 9.07) per meal. We realized the need to acknowledge restaurants that were small enterprises or family enterprises. A total of 87.1% of the participants in this study worked at small businesses; this number represents a general trend across Japan. According to a survey in 2016, 85.3% of accommodation and food service industries in Japan were small businesses [24]; therefore, the results of this study represent the general state of affairs. Considering this, together with the finding that company size was related to higher readiness to take action toward national goals, we infer that small businesses may not have the management policies or management plans that big companies generally have. In addition, large food companies are highly conscious of their corporate social responsibility (CSR) and tend to try to contribute to society; however, they are more likely to invest resources in environmental issues rather than health-related ones [25]. This study also shows that the readiness to contribute toward maintaining healthy weight is lower than that toward minimising food waste. Nearly half of the managers either found it difficult to take action or showed willingness to take action but have not developed a plan to maintain healthy weight; thus, it is essential to specify action plans and details that support this goal, for example, by reducing portion sizes.

This study also indicates that even small business companies, if they were aware of SDGs, were likely to have higher levels of readiness to take action toward both national goals. This represents a positive outcome with respect to small businesses in the restaurant industry in the pursuit of the aforementioned goals. However, over 70% of the participants reported to have ‘never heard about the SDGs’. Although a survey reported 43% of university students were aware of the SDGs [26], this result is not comparable to ours because of the respondents’ widely divergent backgrounds. Future research should study awareness of the SDGs in companies, including the restaurant industry, in other countries.

To motivate restaurants to take action in support of the national goals, some incentive would be needed, especially for small businesses managers. Lindberg et al. [27] conducted a programme for improving the restaurant environment in a rural community. They ranked restaurants into three levels (bronze, silver, and gold) according to the number of healthy practices they have adopted, and offered incentives such as promoting the restaurants via the local media. There is a similar programme in Japan that certifies the restaurants or food service companies which provide menus according to healthy meal criteria [28]. These kinds of programmes would incentivise restaurants to take action toward improving their customers’ health and maintaining a sustainable environment while improving their image as well.

### Strengths and limitations

This study has some limitations. First, because of the nature of self-report questionnaires, there would have existed differences in the participants’ definition of ‘taking action’ to achieve goals. Second, as few participants reported ‘taking action’, we could not analyse the characteristics of restaurant managers who were taking action and could only focus on a level of higher readiness to take action. We acknowledge this as the actual status of the restaurant industry. We found that very few restaurant managers were cognisant of the SDGs or taking action toward national goals. Qualitative methods, such as interviews, should be employed in future research.

Although this study has some limitations, this is the first study to demonstrate the awareness of SDGs in the restaurant industry and its relationship toward taking action in support of the national goals of maintaining healthy weight and minimising food waste, bearing in mind that the respondents were predominantly small businesses. This study indicates that due awareness of the SDGs has not yet penetrated the restaurant industry.

Serving appropriate amounts of food is one way to support both goals of maintaining healthy weight and minimising food waste. However, many chefs believe that the portion size influences customer satisfaction, with large portions implying good value for money [29]. Therefore, measures are needed to encourage customers to request proper serving sizes of food at restaurants, such as by altering the perceived value of meals from being based on the quantity served to other aspects, such as taste. This should equally apply to both restaurateurs and customers. One study showed that interventions from the waiter can affect a customer’s excessive ordering behaviour at restaurants [30]. This suggests that communication between restaurants and customers could be an effective method to induce patrons to select moderate servings of food. Furthermore, it would be important to educate people about taking proper portion sizes and minimising plate waste in the early stages of food consumption behaviour, such as for school meals [31].

## Conclusions

This study revealed that very few Japanese restaurants have initiated actions to contribute to the achievement of the national goals of minimising food loss and waste. These goals are also included in the SDGs. As the SDGs are drawing increasing attention, a corporate responsibility to participate in the achievement of these goals is developing. It is suggested that raising awareness of the SDGs would increase restaurants’ contribution to these goals.

## Data Availability

The data sets used and analysed during the current study are available from the corresponding author on reasonable request.
